# Improvement of obsessive–compulsive disorder after pallidothalamic tractotomy for cervical dystonia

**DOI:** 10.1002/acn3.51764

**Published:** 2023-03-23

**Authors:** Shiro Horisawa, Rumiko Kamba, Moeko Sato, Akitsugu Sueki, Takakazu Kawamata, Katsuji Nishimura, Takaomi Taira

**Affiliations:** ^1^ Department of Neurosurgery Tokyo Women's Medical University Tokyo Japan; ^2^ Department of Psychiatry Tokyo Women's Medical University Tokyo Japan

## Abstract

A 30‐year‐old woman with tardive dystonia in the cervical region from long‐term antipsychotic meds was treated with radiofrequency ablation of the right pallidothalamic tract in the fields of Forel. The patient showed improvement in both cervical dystonia and obsessive–compulsive disorder after the procedure, with 77.4% improvement in cervical dystonia and 86.7% improvement in obsessive–compulsive disorder. Although the treatment site in this case was intended to treat cervical dystonia, the lesion was located in the optimal stimulation network for both obsessive–compulsive disorder and cervical dystonia, suggesting that neuromodulation of this region could potentially treat both simultaneously.

## Introduction

The fields of Forel (FF) is a region in which various nerve fibers, including the pallidothalamic tract (PTT), course and is located caudal to the thalamus, surrounded medially by the mamillothalamic tract, laterally by the subthalamic nucleus (STN), and anteriorly by the red nucleus (RN). Neuromodulation of FF has been reported to improve epilepsy as well as movement disorders, including Parkinson's disease and dystonia, and is noteworthy in that it is possible to neuromodulate multifunctional neural networks simultaneously. Neuromodulation focusing on functional networks is forming the idea that therapeutic effects can be achieved by targeting disease‐relevant network.^1^ Drug‐resistant obsessive–compulsive disorder (OCD) has been reported to improve with neuromodulation of various targets, including the anterior limb of the internal capsule (ALIC), STN, and superolateral medial forebrain bundle (slMFB).^2^ Recently, it was reported that these effective targets for OCD clustered on or around a common functional network.^3^ Therefore, various therapeutic targets produce similar therapeutic effects by neuromodulation of a common functional network.^3^ Cervical dystonia has been treated mainly in the globus pallidus internus (GPi). We have previously reported that radiofrequency thermocoagulation with PTT at the FF for cervical dystonia ameliorates cervical dystonia to the same extent as pallidotomy.^4^, ^5^ We experienced a significant improvement in coexisting obsessive–compulsive symptoms in a patient with cervical dystonia after radiofrequency ablation of the right PTT.

## Case Presentation

**Figure 1:**
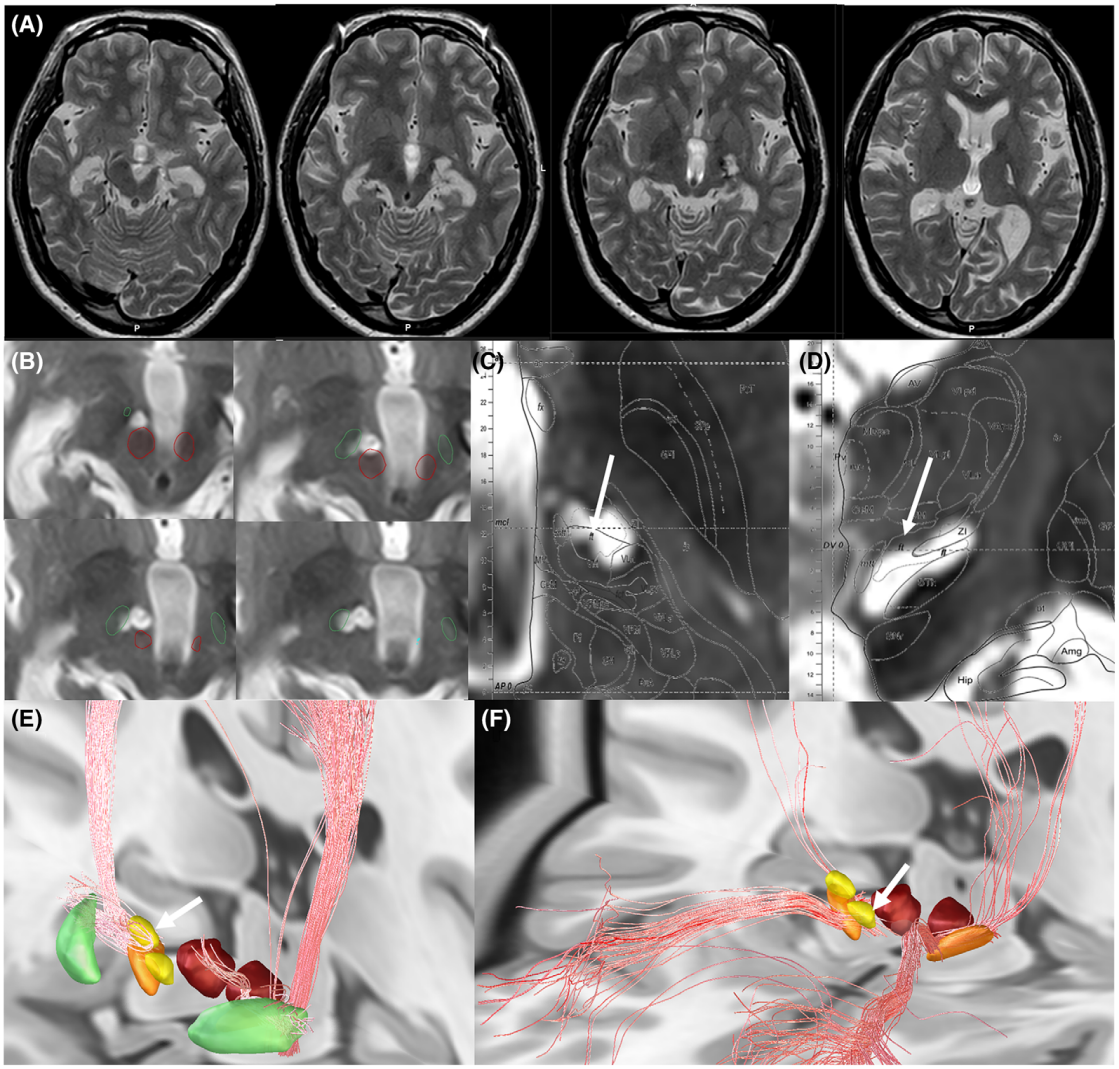
Postoperative imaging analysis. (A) Preoperative axial T2‐weighted MRI showing post‐traumatic lesions in the left hemisphere. (B) 3‐month postoperative T2‐weighted axial MRI showing a medial lesion anterior to the red nucleus and a lateral lesion supero‐medial to the subthalamic nucleus. Green: subthalamic nucleus; red: red nucleus. (C and D) Anatomical location of the pallidothalamic tract at the fields of Forel shown by postoperative axial (C) and coronal (D) left–right flipped MRI overlaying Morel's atlas. White arrow represents thalamic fasciculus (ft) covered by the radiofrequency lesions. The area of the ft is called by fields of Forel H1. (E) Fiber tracts showing optimal stimulation network for cervical dystonia by Lead‐DBS. Lateral lesion (white arrow) is on the fiber tracts. Green: globus pallidus internus; orange: subthalamic nucleus; crimson: red nucleus; yellow: lesions. (F) Fiber tracts showing effective network for obsessive–compulsive disorder by Lead‐DBS. Medial lesion (white arrow) is on the fiber tracts. Orange: subthalamic nucleus; crimson: red nucleus; yellow: lesions.

A 30‐year‐old female patient presented with a history of a motor vehicle accident at the age of 15 years with a cerebral contusion mainly in the bilateral temporal lobes and left globus pallidus leading to the hemiparalysis of the right upper and lower limbs (Fig. 1A). Two months after the accident, behavioral abnormalities, such as incoherent speech, paranoia, aggressive behavior toward others, auditory hallucinations, frequent hand washing, and prolonged bathing, began to appear. She was diagnosed with schizophrenia and OCD after traumatic brain injury by a local psychiatrist and was prescribed olanzapine 5 mg, which resulted in significant improvement of her incoherent speech and paranoia. At the age of 28, she developed symptoms of left lateral flexion of the neck and left shoulder elevation. The patient was diagnosed with tardive dystonia and was treated with medications by neurologists but did not improve; therefore, she was referred to our department for surgical treatment. Since the patient strongly refused the implantation of the instrument, we selected radiofrequency thermal coagulation. Dystonia was localized to the neck and shoulders, and the Toronto Western Spasmodic Torticollis Rating Scale (TWSTRS, range: 0–85, with higher scores indicating greater severity) was 31. About 20–30 hand washings per day were observed, each taking 10–20 min. The daily bathing time was 2 h, and once a week, the patient took a 6 h bath. Auditory hallucinations were reported once daily. She had a sleep–wake rhythm disorder, and her daily rhythm was 24 h awake and 24 h asleep. At the psychiatric department, the Mini Mental State Examination (MMSE, range: 0–30, lower scores indicating lower cognitive function), Yale–Brown Obsessive–Compulsive Scale (Y‐BOCS, range: 0–40; higher scores indicating greater severity of OCD symptoms), Hamilton Depression Rating Scale (HAM‐D, range: 0–54, higher scores indicate greater severity), and State–Trait Anxiety Inventory (STAI)‐ X1 (range: 20–80; higher scores indicating more severe state anxiety)/X2 (range: 0–80, higher scores indicating more severe trait anxiety) were evaluated (Table 1). Radiofrequency ablation of the right PTT was performed for cervical dystonia, which resulted in significant improvement of cervical dystonia. The detailed surgical procedures are reported in a previous study.^4^ Immediately after the surgery, cervical dystonia improved, and mild muscle weakness appeared. The patient was discharged after 2 weeks of rehabilitation. The number of hand washings decreased to less than 10 times a day, and the time spent washing hands decreased to less than 3 min. The bathing time was less than 40 min, and the sleep–wake rhythm normalized, except for insomnia once every 3 weeks. Auditory hallucinations decreased to once a month. Three months after surgery, the patient was evaluated again by psychiatrists for MMSE, Y‐BOCS, HAM‐D, and STAI‐X1/X2 the scores were compared with the previous ones (Table 1). Furthermore, TWSTRS score was 7. Muscle weakness was fully restored to the preoperative status. T2‐weighted axial magnetic resonance imaging (MRI) revealed two lesions medial to the STN and anterior to the RN (Fig. 1B). The anatomical location of the PTT at the FF with Morel's atlas is shown in Fig. 1B,C.^6^ Tractography of the medial and lateral lesions constructed by preoperative diffusion tensor imaging using Brainlab elements (Brainlab AG, Munchen, Germany) showed a projection of both the lateral and medial lesions to the orbitofrontal cortex via the ALIC. Postoperative T1/T2‐weighted axial MRI images were processed with Lead‐DBS (www.lead‐dbs.org) and MRIcroGL (https://www.nitrc.org/projects/mricrogl) to visualize the spatial location of the postoperative lesions, and their relationship to the reported optimal stimulation networks for OCD and cervical dystonia were examined. The medial lesion was located in the optimal stimulation network of the OCD, and the lateral lesion was located in the optimal stimulation network of the cervical dystonia (Fig. 1D,E). At the final evaluation, 16 months post‐treatment, cervical dystonia, and psychiatric conditions were maintained. Despite no significant change in food intake, the patient's body weight increased by 5 kg.

**Table 1:** Pre‐ and postoperative clinical evaluations.

	Pre‐surgery	3‐Month post‐surgery	% Improvement
TWSTRS	31	7	77.4
MMSE	23	24	4
Y‐BOCS	15	2	86.7
HAM‐D	7	5	8.6
STAI‐X1	41	35	14.6
STAI‐X2	45	39	13.3

HAM‐D, Hamilton Depression Rating Scale; MMSE, Mini Mental State Examination; STAI, State–Trait‐Anxiety‐Inventory; STAI‐X1, anxiety as a reaction to episodic stress condition; STAI‐X2, anxiety as a general predisposition for anxious behavior; TWSTRS, Toronto Western Spasmodic Torticollis Rating Scale; Y‐BOCS, Yale‐Brown Obsessive–Compulsive Scale.

## Discussion

In this case, radiofrequency ablation of the right‐side PTT at FF for the treatment of cervical dystonia simultaneously improved not only cervical dystonia (77.4% improvement on TWSTRS) but also obsessive–compulsive symptoms (86.7% improvement on Y‐BOCS). OCD in this patient is thought to be related to head trauma. Obsessions and compulsions have been reported to occur in approximately 30% of patients following head trauma, with mesial prefrontal and temporal lobe lesions being particularly associated with obsessions.^7^ The head MRI (Fig. 1A) shows lesions in the left temporal lobe. Attempts to investigate the functional network contributing to symptomatic improvement by neuromodulation have been reported in OCD and dystonia,^3^, ^8^ and the mechanism by which various treatment targets produce similar symptom improvements is being elucidated. We have previously reported that radiofrequency ablation of GPi and PTT can have similar improvements on cervical dystonia.^4^, ^5^ We preferred to use the PTT rather than the GPi because GPi is adjacent to vital structures, including posterior limb of internal capsule (PLIC) and optic tract, and pallidotomy carries the risk of delayed cerebral infarction on the PLIC.^9^ In contrast, the PTT at the FF does not have surrounding structures that can induce hemiparesis or visual field disturbances. Additionally, there have been no reports of delayed cerebral infarction after PTT surgery. In our radiofrequency ablation of PTT, two lesions were made to interrupt the PTT including ansa lenticularis and lenticular fasciculus. The medial lesion was created anterior to the RN and medial to the STN, and the lateral lesion was created posterolaterally to the medial lesion and above the STN.

OCD has been reported to have various therapeutic targets for neuromodulation, including the ALIC, nucleus accumbens, anteromedial GPi, STN, and slMFB.^2^ Li et al. reported that these effective targets for OCD clustered on or around one common functional network that connects the frontal regions to the STN.^3^ This functional network is available as an atlas in stereotactic space in Lead‐DBS software. The medial lesion in the present case was located within this network (Fig. 1D). Additionally, Meyer et al. reported the slMFB as an effective deep brain stimulation (DBS) target for OCD.^10^ They reported that the electrode location of the slMFB DBS was anterior to the RN and medial to the STN, which is consistent with the site of the medial lesion in our case.

GPi has been used as a therapeutic target in cervical dystonia. In GPi‐DBS for cervical dystonia, an optimal stimulation network has also been reported based on the stimulation sites that contribute to symptom improvement.^8^ This network is also available as an atlas in the Lead‐DBS software. In this case, the lateral lesion was located in the optimal stimulation network for cervical dystonia (Fig. 1E). Improvement of cervical dystonia by STN‐DBS has also been reported, with stimulation of the dorsal STN being effective.^11^ The lateral lesion in this case was located above the STN and may have partially ablated the dorsal region of the right STN.

Interestingly, only the right‐sided ablation of PTT improved OCD symptoms. Among patients who underwent bilateral anterior capsulotomy for OCD, those with only the right‐sided lesion within the anterior capsule were commonly good responders with a Y‐BOCS improvement of more than 50%.^12^ DBS of the right ventral capsule/ventral striatum has recently been reported to be effective in depression.^13^ The left and right hemispheres may not always be equally involved in the pathogenesis of psychiatric disorders. The question of whether there is a dominant hemisphere in the development of psychiatric disorders is interesting and needs to be clarified to establish more effective treatments.

Although the treatment site in this case was intended to treat cervical dystonia, the functional network involved in OCD may also run in the caudal region of the FF. Targeting this region could potentially simultaneously treat dystonia and OCD.

## Funding Information

This work was supported by JSPS KAKENHI Grant Number JP21K09113 and Japan Brain Foundation.

## Conflict of Interest

None.

## Author Contributions

Shiro Horisawa, Katsuji Nishimura, Takakazu Kawamata and Takaomi Taira contributed to the conception and design of the study. Shiro Horisawa, Rumiko Kamba, Moeko Sato, and Akitsugu Sueki contributed to acquisition and analysis of the data. SH drafted the text and prepared the figure. All authors approved the final version of the manuscript.
